# PLD plasma plume analysis: a summary of the PSI contribution

**DOI:** 10.1007/s00339-023-06408-4

**Published:** 2023-01-21

**Authors:** Christof W. Schneider, Thomas Lippert

**Affiliations:** 1 Laboratory for Multiscale Materials Experiments, Paul Scherrer Institute, 5232 Villigen PSI, Switzerland; 2 Department of Chemistry and Applied Biosciences, Laboratory of Inorganic Chemistry, ETH Zurich, Zurich, Switzerland

**Keywords:** Pulsed laser deposition, Thin films, Plasma spectroscopy, Mass spectrometry, Kinetic energy distribution

## Abstract

We report on the properties of laser-induced plasma plumes generated by ns pulsed excimer lasers as used for pulsed laser deposition to prepare thin oxide films. A focus is on the time and spatial evolution of chemical species in the plasma plume as well as the mechanisms related to the plume expansion. The overall dynamics of such a plume is governed by the species composition in particular if three or more elements are involved. We studied the temporal evolution of the plume, the composition of the chemical species in the plasma, as well as their electric charge. In particular, ionized species can have an important influence on film growth. Likewise, the different oxygen sources contributing to the overall oxygen content of an oxide film are presented and discussed. Important for the growth of oxide thin films is the compositional transfer of light element such as oxygen or Li. We will show and discuss how to monitor these light elements using plasma spectroscopy and plasma imaging and outline some consequences of our experimental results.

**Supplementary Information:**

The online version contains supplementary material available at 10.1007/s00339-023-06408-4.

## Introduction

The preparation of oxide thin films using pulsed high power lasers has advanced considerably since the discovery of the cuprate superconductors [1, 2]. With the cuprates being typically multi-elemental, the appeal was then and still is that complex multi-elementary materials can be deposited from a preferentially single-phase, homogeneous target [3–8]. In addition, the source to remove the material from the target is outside the vacuum chamber, which gives a certain degree of freedom with the design of the deposition chamber. A drawback of pulsed laser deposition (PLD) compared to most other physical vapour deposition techniques is the small size of substrates, which can be homogeneously covered, typically 10 × 10 mm due to its very directional deposition character. For the typical applications of thin film preparation by PLD within a research context, the substrate size is not that important. For large-scale deposition, however, different solutions have been successfully implemented [9–11]. One unique point for laser ablation is the pressure range at which films can be prepared ranging between the base pressure of the vacuum system up to mbar and its relative ease using reactive background gases to help the chemistry to prepare oxide thin films with a complex composition. This also makes it clear that PLD is on the one side a very versatile deposition technique, and on the other side intrinsically more complex than most think. For many, PLD is a “black box” delivering easily a complex composition of the growing film.

To show how far we have come in our understanding, we will describe in this contribution how the elemental distribution in an expanding laser-induced plasma plume looks like and how this is transferred to a film including the issue of transferring light elements. Techniques used and reported here to study these processes are energy-resolved mass spectroscopy, time, space and species-resolved optical imaging [12–15], and secondary ion mass spectroscopy (SIMS) to study the composition of thin films. We further highlight the importance of negative ions in the plasma for the growing film. A relevant topic is the source of oxygen for a film (target, background gas, substrate). At the end, we will present more recent results on the plasma dynamics of target materials with a multi-elemental composition.

## Experimental

The ablation experiments were performed in an ultrahigh vacuum (UHV) chamber (base pressure 1 × 10^−9^ mbar) using KrF and ArF excimer lasers (Lambda Physik LPX 300, 20 ns pulses, *λ* = 248/193 nm) and an XeCl excimer laser (Lambda Physik LPX 205, 20 ns pulses, *λ* = 308 nm). To define the laser beam profile, a square aperture is imaged with dimensions of 1 × 1 mm on cylindrical as well as disk-shaped ceramic targets [16, 17]. The laser pulses arrive at the target at an incidence angle of 45° with respect to the target–substrate (TS) axis. To avoid cratering, the target was continuously displaced and rotated. Laser fluences between 1 and 3.5 J/cm^2^ were used and the background gas pressure varied between vacuum (< 1 × 10^−6^ mbar) and 0.2 mbar. As background gas, O_2_, N_2_O and Ar have been used [16–18].

The plasma plume ions were analysed using an ion-energy analyser combined with a quadrupole mass spectrometer (MS) from Hiden Analytical Ltd. (HAL EQP) capable of measuring ions with kinetic energies up to 1000 eV, using a 45° sector field. It is installed in the same UHV chamber with its 30 µm orifice aligned along the plasma plume expansion axis. The typical target-to-MS distance was 40 mm for most experiments used, but also varied up to 90 mm. The MS measurements are gated using an adjustable Stanford Delay Generator DG535 triggered by the laser beam through a photo-diode. Typically, a delay of 100 ns and a gate width of 500 μs were used. At the distance of 40 mm, the plasma expansion is observed during a period of approximately 5–150 μs. The maximum kinetic energies were defined for signals at which the counts detected exceeded more than five times the noise levels [16–18].

For the optical measurements, an intensified charge-coupled device (ICCD) “Andor New i-star” DH334T-18–03 was used to record the time evolution of the emitting species of the ablated material. The sensor has a spectral range from 180 to 850 nm, a 1024 × 1024 pixel size, and the relationship between image pixels and physical distance was calculated using the reference distance of 4 cm. The images were recorded for all light passing through a quartz window (200 nm–1000 nm) or for selected wavelengths using an Acousto-optic tuneable filter (AOTF, 400–1000 nm). The AOTF (Brimrose models VA210-0.55–1.0-H and VA210-0.40–0.65-H) has a wavelength resolution of 0.6–3 nm with a manufacturer-certified resolution of 1.3 nm at 633 nm. In both cases, a 28–300 mm f/3.5–6.3 macro lens was mounted on the ICCD and set at f/3.5 or f/5.6 for the AOTF measurements [16–18].

The camera was triggered externally by the laser pulse using a photo-diode. The onboard digital delay generator was used for gating the image intensifier and increasing time delays were used to capture its time evolution. Typically, a gate width of 5 ns for the plume expansion below 2.5 ls and 50 ns for later times were used, while for the AOTF measurements a gate width of 100 ns below 2.5 ls and 1000 ns for all other measurements were applied. A gain value of 3500 was used for all non-AOTF and 4095 for all AOTF measurements. For each time frame, an accumulation of 40 images was used to improve the signal-to-noise ratio [16–18].

For the elemental composition, Rutherford backscattering spectrometry (RBS: with 2–4 MeV ^4^He ions) or elastic recoil detection analysis (ERDA) was used depending on the target material. Software from the Ion Beam Physics group at ETH Zurich and the software package RUMP were used for data analysis [19].

## Plume properties

### Laser-induced plasma plume

For laser ablation to work, two basic requirements are necessary [20, 21]. For one, the target material must absorb the laser wavelength sufficiently well. For oxide ablation, this is best in the UV energy range. Second, the laser fluence must be large enough to exceed the ablation threshold which is for oxides typically of the order 1 J/cm^2^ [20, 22–25]. Alternatively, the pulse length can be varied from ns down to fs since the removal mechanism also varies with the time scale of the solid–laser interaction [26–28]. If the fluence is below the threshold, the target material is partially ejected and no plasma is formed. For an ablation above the target material’s fluence threshold, three processes simultaneously take place: thermal, photophysical and photochemical ablation. The first one leads to a rapid temperature rise, easily reaching up to 10,000 K. Non-thermal interactions dominate the initial ablation such as photo-excitation of an electron or multiphoton-absorption processes and the combination of both processes contributes largely to the formation of a plasma plume. The photochemical ablation is mainly responsible for direct bond breaking of molecules, which explains why this is a relevant process as long as the rapidly expanding plasma plume interacts with the incoming laser light. These interactions therefore set a time scale for the laser–solid interaction [27, 28]. For femtosecond pulse durations, typical time scales of the electron dynamics are similar to that of the pulse duration and thermal effects can be neglected. Here, an extreme non-equilibrium state is created living up to a couple of picoseconds. This state is characterized by the presence of very hot electrons that, after absorbing a large number of photons from the laser pulse and electrons thermalizing through electron–electron collisions, acquire an extremely high temperature. Overall, the lattice remains cold, since the laser excitation and electron thermalization occur on a time scale much shorter than lattice heating through incoherent electron–phonon coupling. The removal of material, however, proceeds via a so-called Coulomb explosion which is equivalent to an overheating of the irradiated material. The resulting plume consists mostly of nano-scaled particles and not as much of atomic species. For a pulse length shorter than approximately 10 ps, photophysical and photochemical processes will dominate, as the interaction time with the laser beam is comparable with the timescale for the initial plume formation. Also, thermal effects are limited since the interaction time is similar to the electron–phonon coupling time. As the pulse length increases to 100 ns and above, the ablation will be driven primarily by thermal effects and expulsion of the melt will dominate including liquid droplets being ejected. This mixture of thermal and photochemical processes for the typical excimer laser pulse length of 20 ns or the 8 ns for Nd:YAG lasers as used today proved to be the best combination when preparing high-quality oxide thin films.

### Plasma expansion

**Fig. 1:**
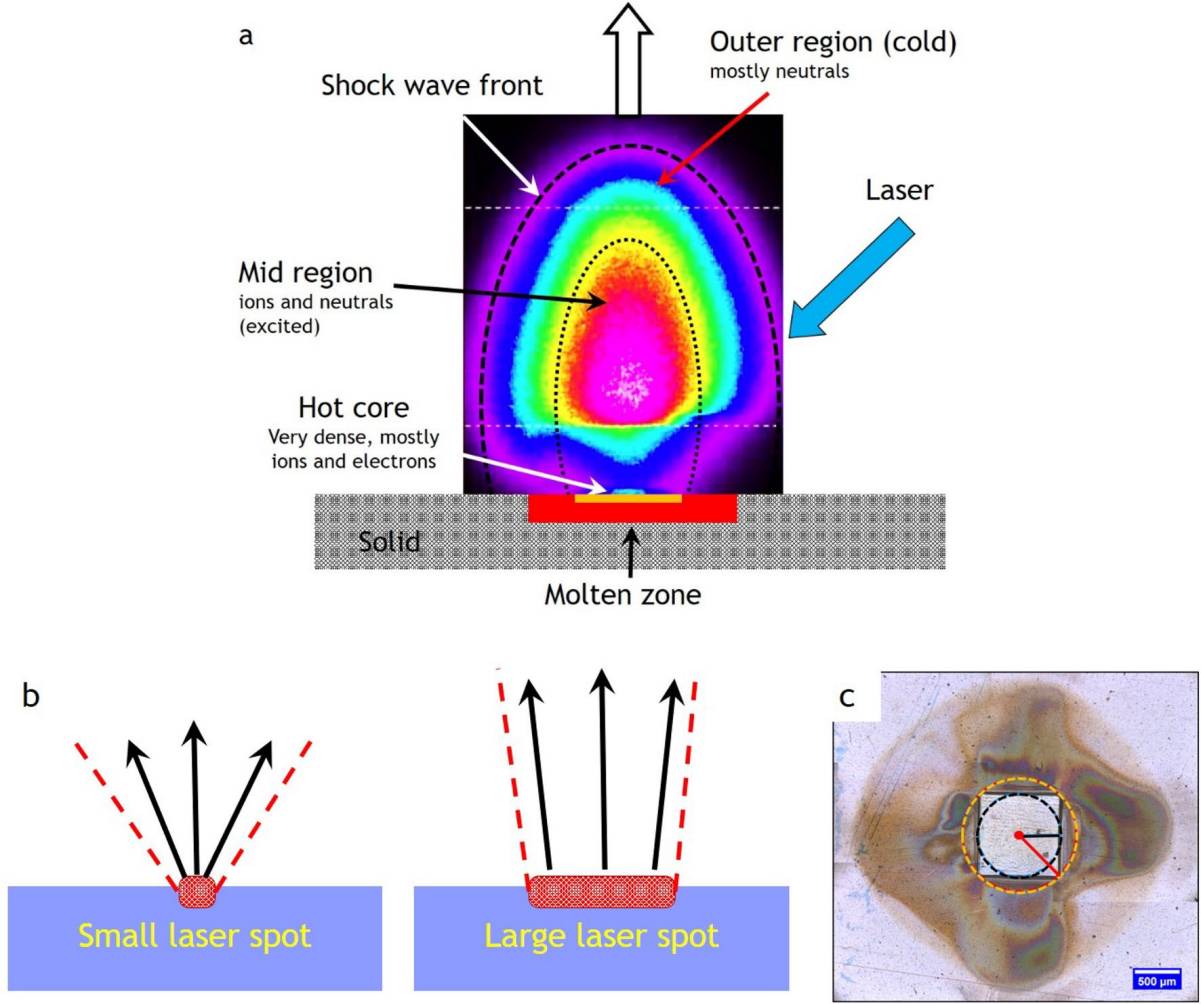
**a** Illustration of the different zones of an expanding laser-induced plasma plume. **b** Schematic showing the changes in the opening angle of the plasma plume based on the spot size dimensions. **c** Room temperature ambient ablation of polyimide using a square beam profile. The recondensed ablation fragments show the effect of the longest and shortest axis on the material distribution associated with the flip-over effect [16, 45]

The striking feature of PLD is the laser-induced plasma plume consisting of (excited) neutral and ionic species and electrons, which move at a very high velocity from the target to the substrate [12, 14, 29–32]. For a typical TS distance of 4 cm, this takes in vacuum between 1 and 2 µs at the often-used deposition pressure in the 0.1 mbar range of the order 20–30 µs. An image of a plasma plume is shown in Fig. 1a, also illustrating the different regions which develop during the expansion giving rise to a very complex plume dynamic. With one laser pulse and a fluence of 1–2 J/cm^2^, typically 10^21^–10^22^ atoms are removed from a depth of a couple of 10 nm, but in general the ablation depth is fluence dependent and varies accordingly [33, 34]. In this hot core region, a nearly neutral vapour is ejected forming a thin vapoor layer above the target surface [35–39]. The subsequent plasma generation takes place via optical breakdown, which is favoured by a large density of free electrons from the initially laser-generated vapour [35, 36, 40]. The electrons ejected from the surface originate either from the photoeffect and those formed due to the ionization of atoms. The laser-generated electrons are accelerated away from the vapour layer forming an electric field gradient with the ions via an electric double layer [35, 36, 41], an electric field in which the subsequently created ions are also accelerated gaining their often-observed large kinetic energy [28, 41, 42]. Here, the maximum kinetic energies of ionic species in the ablated plasma are strongly dependent on their masses, with higher masses yielding higher kinetic energies [41]. The initial hemispherical movement away from the hot core region is quickly elongated with the perpendicular to the substrate surface direction being the major direction [35, 36]. This is also a consequence of the momentum transfer the chemical species experience, with light elements being the faster species and the heavier element the slower species. The elongation becomes very pronounced if elements with very different mass ratios are ablated from the target.

The initially very dense vapour is responsible for having a high degree of ionization and excitation of plasma species due to elastic scattering. This cloud of electrons and ions expands rapidly, thereby forming, at the plasma front, a shock wave zone depending on the vacuum conditions. For a low-pressure regime (< 10^–2^ mbar), there is minimal interaction between the plume and the residual background gas due to a mean free path (mfp) much longer than the mfp inside the plume and the plume species experiences an adiabatic expansion. In a pressure window around 10^–2^ mbar, the so-called transition regime, there is already an increase in interactions between the plume and the background gas leading to plume splitting (two fractions of the same species with clearly different velocity distributions) and shock wave formation [43, 44]. The latter is the result of a slowing down of species due to scattering of the expanding plume front with the background gas leading. In the pressure regime starting around 0.1 mbar, labelled the diffusion-like regime, the formed dense shock wave front is responsible for slowing down the kinetic energy, *E*_kin_, of the plasma species considerably (< 10 eV) and enables a large number of scattering events with additional chemistry to take place inside the plume depending on the reactivity of the background gas. The detected *E*_kin,max_ are different for different target materials and increase with laser fluence [14, 41]. The equilibration of plasma species due to the formation of the shock wave front has an additional effect, that a homogeneous thickness and composition can be achieved, although the film composition is not necessarily identical to the target composition.

The laser spot size has an effect on the plume appearance with a smaller spot resulting in a larger opening angle and a larger spot, leading to a more confined plume (see Fig. 1b) [46–48]. This is similar to the distinction between a point and a surface source for thermal evaporation, with the point source resulting in a more homogeneous thickness distribution. Therefore, a decrease in spot size results in a stronger broadening of the thickness profiles for mono-elemental targets and a directional inhomogeneity of the composition for multi-elemental targets [16, 45]. The geometrically related directional inhomogeneity is an important issue for ablation, since the beam profile of an excimer laser is rectangular with a typical aspect ratio of 1:2, which will naturally lead to a plume species inhomogeneity arriving at the substrate position due to the directional spreading of species [16, 45]. Ideally, a circular aperture should be used because it would avoid the geometrical differences in the expansion of the plume and potentially avoid compositional variations. For practical reasons, a square or rectangular aperture is used to define the beam profile on the target with the effect that the plume will preferentially expand perpendicular to the edges rather than in all directions as shown in Fig. 1c. The effect as visualized in Fig. 1c is known as the flip-over effect [49, 50], but the term “crowd effect” would be more suitable since the spatial distribution of ejected material is strongly affected by the amount of material removed from the target [49, 50]. Since the “crowd effect” is amount (fluence) dependent, the local plume density can vary accordingly and therefore lead to a significant angular dependence inside the plume [16]. The flip-over effect is therefore responsible for compositional variations in films, the main reason being the combination of geometrical effects of the expanding plasma and different scattering properties of plasma species inside the plume [45]. For a small area deposition, compositional variations are usually not a problem. For large area, this fact is certainly relevant.

### Plasma expansion of light elements

**Fig. 2:**
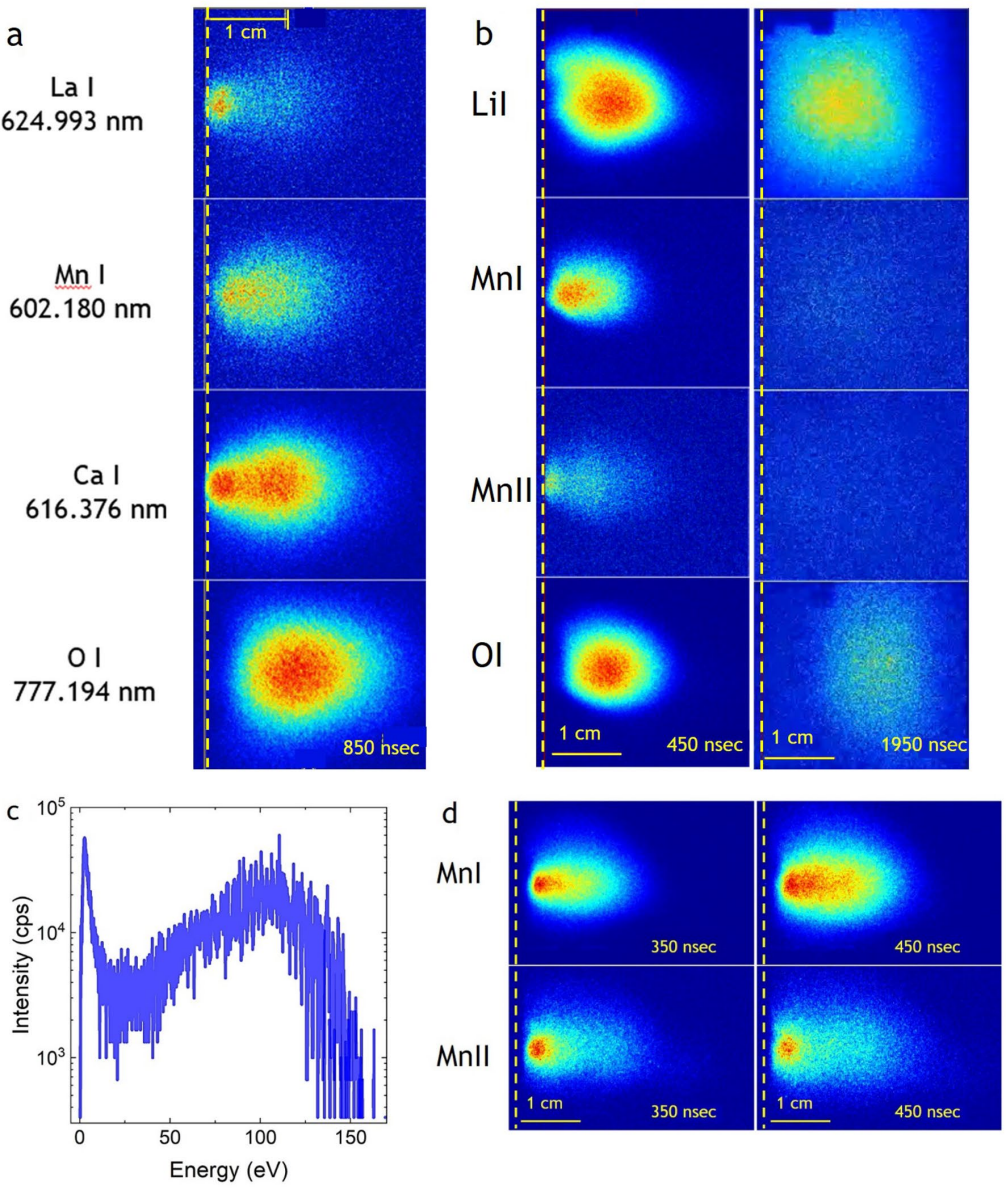
**a** Spatially resolved images of excited La, Mn, Ca and O species 850 ns after laser impact on an La_0.6_Ca_0.4_MnO_3_ target in vacuum and fluence of 2 J/cm^2^ [51] **b** Spatially resolved images of Li I, Mn I, Mn II and O I species 850 and 1950 ns after laser impact on an LiMn_2_O_4_ target in vacuum and a fluence of 2 J/cm^2^ [51]. [51] **c** Kinetic energy distribution for O^+^ for a vacuum ablation of La_0.6_Ca_0.4_MnO_3_ (KED [17]). **d** Spatially resolved images of Mn I and Mn II species 350 and 450 ns after laser impact on an LiMn_2_O_4_ target in vacuum and a fluence of 3 J/cm^2^ [51]

Figure 2a illustrates the effect of momentum transfer for the ablated species. Here, excited state species of neutral La, Mn, Ca and O (La I, Mn I, Ca I, O I) are shown as ablated from an La_0.6_Ca_0.4_MnO_3_ target in vacuum with a fluence of 2 J/cm^−2^ 850 ns after the initial ablation [17]. As expected, the heavy La is slowest and preferentially forward directed, whereas the lightest element, oxygen, is fastest but also more scattered and therefore shows space-wise a much wider distribution. Mn and Ca have a similar mass (*m* = 40, 55) and hence travel with a similar speed with Ca, being faster than Mn. Interesting is the fact that the heavier/heavy element distributions show two fractions, one which is very fast and one with a high intensity being relatively slow. Oxygen, on the other hand, shows only one broad distribution. The interpretation of this twofold distribution is that the fast species are the ones released within the pulse duration of the laser light and the ionized species are accelerated within the aforementioned electric field gradient. The measured neutral excited species are the result of an electron recapture, thereby forming neutral (excite) species travelling at a similar, but on average slower velocity than the ionic species. Once the pulse is terminated, more species are still released from the melt while cooling down and hence these species only experience a partial acceleration in this electric field gradient. As a consequence, they have significantly smaller kinetic energies. This point will be picked up and discussed in more detail in the discussion about the plasma dynamics of multi-elemental plasma plumes. The clear two plume fractions for Ca are the result of a larger vapour pressure for Ca as compared to Mn and hence Ca is more easily released from the melt. As a consequence, the Ca/Mn ratio in the near-surface region is shifted towards Mn and hence the initial target composition will be different compared to the initial target composition. Slow fractions are also observed for Mn and La, which indicates that the density of released slow species is sufficiently large that excited state species can be created via scattering.

Measuring the kinetic energy distribution (KED) for O^+^ under the same ablation conditions (Fig. 2c), the distribution shows a narrow low energy peak and the large majority of ionic oxygen species have kinetic energies larger than 40 eV. Comparing the conclusions drawn from the O^+^ KED with the time and spatial evolution for O I, one can conclude a reasonable similarity between both plasma species with respect to their KE. Monitoring the ionic excited species as shown in Fig. 2b, d for Mn II and comparing the temporal and spatial evolution to Mn I, the velocity profiles are similar and hence their KED will be similar and for a qualitative comparison applicable. Their numbers are probably different. The spatial intensity distribution for Mn I and Mn II at 350 and 450 ns, shown in Fig. 2d, is noticeably different. The ionic excited Mn species stem in large numbers from the point of laser impact on the target and are faster than Mn I species. However, there are not as many Mn II as compared to Mn I and they are more forward directed as one can expect if these species are accelerated in an electric field as discussed in Sect. 3.2.

There are many very fast oxygen species reaching the substrate site, but oxygen is still measurable even several hundreds of ns after the measurable La I signal disappears. Having measured such a large quantity of highly energetic oxygen species poses the question how many of the available oxygen is useable to be included in the formation of an oxide film. Species arriving at a substrate are either adsorbed, migrate on the surface and then chemisorb, desorb or bounce directly, with highly energetic species more likely to bounce off a surface rather than sticking to it. The desorption rate is enhanced for species with a small probability to be adsorbed or arriving at a surface with an elevated temperature. This would qualitatively explain why oxide thin films grown at a low pressure using a 248 nm laser are often oxygen deficient, whereas oxide films grown using a 308 nm laser can have an almost correct oxygen content [52].

Whereas for oxygen-deficient thin film oxygen can often be supplemented by a post-annealing step preferably in situ, the case for the deposition of Li-containing films is more complex. Preparing Li-containing films with a correct composition is difficult even if an over-lithiated target is used. Measuring for a vacuum deposition of LiMn_2_O_4_ the KED for Li^+^ with a fluence of 2 J/cm^2^, we find like for O^+^, an *E*_kin_ maximum at low energies (4–5 eV), but also a distinguished broad distribution between 40 and 100 eV with the large majority of species being highly energetic [17]. Imaging the time and spatial evolution of Li I shows a similar pattern as for oxygen. The initially ablated Li I is significantly faster than O I and Mn I and forward directed, followed by a quick broadening of the Li cloud, due to scattering with other plume species in the same time window. Like for oxygen, Li I can be detected more prominently late in the plume expansion even after the detectability of the oxygen and Mn is very low or below the detection limit of the CCD detector (Fig. 2b). The origin of the detectability of O I and Li I at the very late stage of the plume expansion, which coincides already with the time frame that many of the target species have reached the substrate position is at present an open question. If it would be a simple forward motion of species with a finite lifetime for the detected excitation and a decrease in the scattering rate, it is expected that the recorded intensity becomes smaller with increasing time. The images suggest on the contrary a stationary behaviour for the re-excitation. Knowing that the sticking coefficient on a substrate for Li and O is low and both have large kinetic energies, this would speak for a deflection or rebound of both species from the substrate position, thereby creating a backflow which helps to create sufficient numbers of excited species to be detected well after the average arrival time for plume species at the substrate position. The existence of such a backflow is shown and an example is given in Fig. S1 of the supplementary material [17, 53, 54]. To overcome the issue with a nearly correct Li composition for a film, these experiments show us essentially two options. For one, we have to work preferentially at an elevated pressure to slow down most of the fast Li species. This seems to work only partially with respect to the film stoichiometry. The second option is to increase at the same pressure the TS distance and therefore reduce the number of fast species arriving at the substrate site. That the latter approach works satisfactorily has been shown when depositing stoichiometric Li_*x*_La_*y*_Sr_*z*_MnO_3_ thin films at large substrate–target distances [55]. More work in this direction is necessary to better explore this option including a distance–pressure dependence.

### Plasma plume composition

**Fig. 3:**
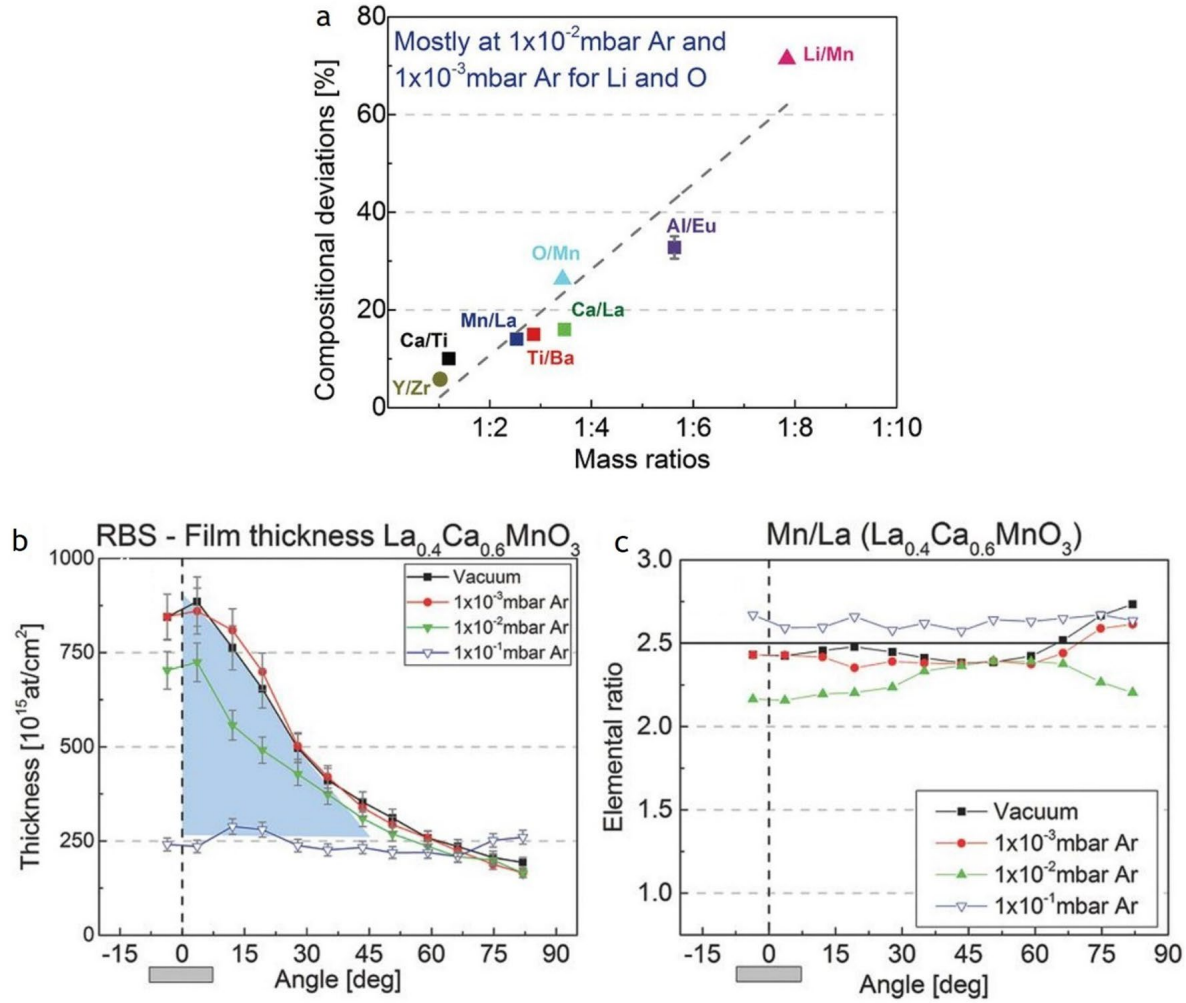
**a** Maximum compositional deviations at PLD-relevant angular areas (± 10°) versus mass ratios, mainly for 1 × 10^−2^ mbar Ar, except for O/Mn and Li/Mn in which the maximum deviations already appear at 1 × 10^−3^ mbar Ar. Symbols: black medium square are for RBS measurements, black medium triangle for ERDA measurements, and black medium circle for data from ref. [59]. Note: O/Mn deviation has a high uncertainty due to the suspicion of ambient water trapping by the amorphous film. Error bars are smaller than the symbols except for EuAlO_3_. Reproduced with permission from ASS Copyright 2016, Elsevier. **b** Angle-resolved film thickness profiles of La_0.4_Ca_0.6_MnO_3_ for vacuum and three different Ar background gas pressures from ref. ASS. The peaked shape of the thickness profile disappears at 1 × 10^−1^ mbar Ar, highlighted in blue. Angle-resolved film composition of **c** La_0.4_Ca_0.6_MnO_3_ for vacuum and those same three Ar background gas pressures. Film thickness and composition were obtained by RBS. The horizontal black lines represent the RBS-verified target composition. **a** Reproduced with permission from JAP Copyright 2017, American Institute of Physics. **b** Reproduced with permission from ASS Copyright 2016, Elsevier.

In the previous section, we have shown that the species distribution of a multi-elemental plume will depend on the respective mass ratios of the target species as well as the pressure at which the deposition takes place. In particular, if the mass ratio is large, it become increasingly more difficult to achieve the correct film composition [55, 56]. The opposite seems to be correct if a material is deposited containing elements with similar masses [57]. To some extent, it will also depend on the kind of background gas. There is a strong relationship between the atomic mass ratios and the compositional deviations in the film. This effect is shown in Fig. 3a for LiMn_2_O_4_ (Li/Mn mass ratio of 1:8) with deviations of up to  ≈ 70% relative to the target composition. These compositional deviations are largest at the highlighted pressure of 1 × 10^−2^ mbar, while at a higher or lower pressure deviations these can be reduced or even minimized [58].

A deposition with a TS distance shorter than the mfp is called a “vacuum-like” deposition with only a few background gas interactions. Intraplume collisions take place and in the case of multi-element materials, the different plume species ions and neutrals of the same element travel with very high, but dissimilar velocities, and as a consequence have dissimilar arrival times at the substrate. Since the release of material is spread over time, the time window of a laser pulse, the probability of scattering events taking place between fast and slow species, is enhanced and will determine to a large extent the dynamics of the plume expansion. In this deposition regime, most of the species are concentrated at the centre of the plasma plume, producing films which are considerably thicker in the vicinity of the plume expansion axis (Fig. 3b). The film composition (black line, square symbols in Fig. 3c) is not necessarily the same as the target, represented by a horizontal line at a value of the elemental ratio of 2.5 in Fig. 3c, but is often similar in the most relevant angular range. The pressure regime 2.5 × 10^−3^ mbar to 5 × 10^−2^ mbar, defined as the “transition” regime, is the pressure window in which the largest film compositional deviations happen, i.e. the Mn/La ratio of films produced at 1 × 10^−2^ mbar Ar as shown in Fig. 3c. It is a pressure range where a certain level of interaction between plasma species with the background gas takes place, and the film thickness varies little as compared to a vacuum deposition. The underlying reason is the preferential scattering of the lighter elements with the background gas, while the heavier elements are less affected. For a deposition pressure larger than 5 × 10^−2^ mbar, it becomes easier again to regain control of the film composition in particular if the mass ratios of the plasma species are not too dissimilar [58, 60].

### Influence of negative ions on plasma properties

Next, we will discuss the contribution of negative ions to the plume composition and subsequent film growth. In Sect. 3.1, we mention that the plasma as created with a 248 nm laser of a 20 ns pulse width consists mainly of atomic species. In addition, there are also some binary and tri-atomic species. The latter are rare and measured in small quantities only. In a classical plasma, normally only positive ions are found. Negative ions can exist and are created via an electron attachment to neutral plasma species. However, the electron can be recaptured in a collisional event. To measure negative species, the plasma density needs to be sufficiently low, which can only be at a time well after the laser pulse has terminated and the plume reached a certain expansion. However, measuring negative ions in a laser-induced plasma cannot be taken for granted [61] and is subject to the creation of plasma species depending on the laser wavelength, fluence or plume species density used.

**Fig. 4:**
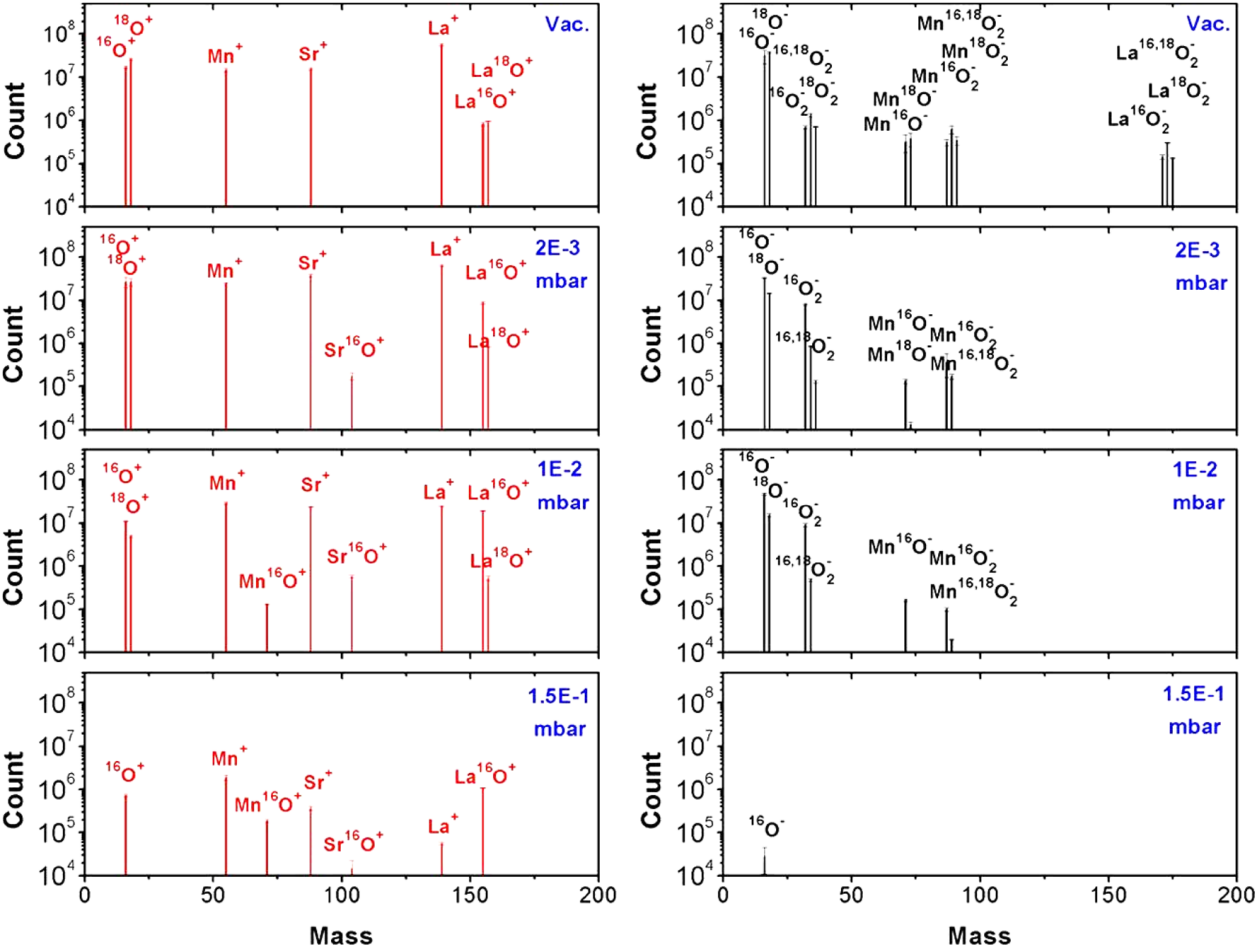
Mass spectra of positive and negative plasma species ablated from an ^18^O-substituted La_0.6_Sr_0.4_Mn^16,18^O_3_ target at four different oxygen background gas conditions (vacuum, 2 × 10^–3^ mbar, 0.01 mbar and 0.15 mbar) with a fluence of 2J/cm^2^. (from [62], Fig. 1b)

Figure 4 shows the mass spectra of positive and negative plasma species as ablated from an ^18^O-substituted La_0.6_Sr_0.4_Mn^16,18^O_3_ target at four different oxygen background gas conditions (vacuum, 2 × 10^–3^ mbar, 0.01 mbar and 0.15 mbar; *λ*  = 248 nm, *Φ* = 2 J/cm^2^) [52]. The ^18^O exchanged target was used to investigate the origin of oxygen in a film and to trace negative ions [52, 62]. For the vacuum ablation, only positive ions and neutral atomic species can be measured except LaO species [18, 52, 61]. This is similar when ablating LuMnO_3_ with a wavelength of 248 nm [18]. The almost equal quantities of ^16^O and ^18^O indicated an isotope substitution in the target of about 50%. With increasing oxygen background pressure, more metal–oxygen (MO) species can be measured and the ratio between ^16^ and ^18^O changes in favour of ^16^O. At the highest pressure measured (0.15 mbar), no ^18^O can be detected, as ion or La^18^O. The most likely reason is a dilution effect. For one, the amount of ^18^O provided by the target is small compared to the number of background species. The increased dissociation/creation rate with increasing pressure plus the enhanced scattering rate for oxygen makes it not very likely to detect many if any ^18^O species at an elevated pressure.

The situation is different for negative ions. There, next to atomic species, also binary and tri-atomic species can be measured including the fully or partially ^18^O-substituted species. The number of binary and tri-atomic negative species decreases significantly with increasing oxygen pressure until about 0.15 mbar where only some O^−^ can be detected. Measuring positive and negative plasma species using a wavelength of *λ* = 308 nm with *Φ* = 2 J/cm^2^, the spectra for the different pressures for both charged species will not look too different except that at the highest pressure no O^−^ can be detected [52]. Exchanging oxygen with the more reactive N_2_O as background gas, more O^−^ species at a higher pressure are measured as expected [18, 52, 61]. Using a 193 nm laser, again, the overall appearance of the mass spectra is similar to that presented in Fig. 4 [61], indicating that changing the photon energy from 4 to 6 eV does not change significantly which species are measured as part of the laser-generated plasma plume. However, a wavelength of 193 nm a more reactive background gas like N_2_O seems to be much more effective in creating negative plasma species as shown in Fig. 2 in reference [63], which also reflects the kind of neutral species present in the plume.

How can we understand the presented spectra for positive and negative ions? The ablation was done with a photon energy of 5 eV (*λ* = 248 nm), and a very typical binding energy range for many MO species is between 2 and 5 eV. Hence, when measuring for positive ions, largely a mono-atomic mass spectrum for a vacuum ablation with *λ* = 248 nm is expected. In addition, measuring La^16/18^O is equally expected, since the binding energy of LaO is 8.2 eV and only a two-photon absorption or elastic scattering providing enough energy to dissociate LaO^+^ into La^+^ and O^+^ can account for the presence of La^+^ and LaO^+^ in the same spectrum. This means that most of the detected LaO species originate directly from the target, since the ^16/18^O ratio is what we expect from the isotope exchange procedure. With increasing oxygen pressure, more MO species will be formed than dissociated due to chemical reactions inside the plasma plume as can be seen with the increasing number of SrO^+^ species with increasing pressure (Fig. 4). Remarkably, newly formed M^18^O cannot be detected, which means that these binaries are formed with the oxygen provided by the background gas as one could expect, since the number of ^18^O coming from target is small compared to the number of oxygen background species.

More interesting is the situation for the negative ions, since they are directly formed from neutral plume species as a direct consequence of the ablation process and an electron attachment to neutral species. Whereas neutral MnO, MnO_2_ and LaO_2_ must exist in the gas phase, SrO_2_ seems to be not stable enough. Other noticeable absentees in all spectra are LaO and SrO as well as elemental La and Sr, which means no electron attachment can be done for both, at least not with the plume dynamic created using a 248 nm laser and a fluence of 2 J/cm^2^, and no O_2_^+^ can be formed. O_2_ rather dissociates directly to atomic oxygen instead of being a positive ionic molecule. A probable reason for the absence of these species is their electron affinity as listed in Table 1 [52, 64]. Increasing the fluence or using a wavelength of *l* = 193 nm seems to stabilize these species [18, 52, 63, 65].

**Table 1:** Electron affinity data for species shown in Fig. 4 [52, 64]

Species	O	Mn	Sr	La	O_2_	MnO	SrO	LaO
Electron affinity (eV)	1.46	− 0.50	0.05	0.56	0.45	1.37	0.64	1.00

How useful or even necessary is the presence of O^−^ in the plasma plume during the ablation process to prepare thin films with the desired properties? This question is difficult to evaluate for most of the materials we prepare, because a deposition process is multifactorial and to single out one parameter is often not possible. For two examples, however, it was possible to show explicitly the positive influence of O^−^ on growth properties. The first example is the growth of La_0.6_Sr_0.4_MnO_3_ films on (100) SrTiO_3_ using a 193 nm laser, oxygen and N_2_O as background gas with a fluence of 1.5 J/cm^2^ at *p*_N2O/O2_ = 0.15 mbar [63]. The use of a shorter wavelength and a more reactive background gas (N_2_O or O_2_) provided a total yield of negatively ionized species of 24% (12% and 14% in O_2_ and vacuum, respectively) and positively ionized species of 36% (55% and 36% in O_2_ and vacuum, respectively). As a result, the composition of the as-grown film (without O_2_ annealing) was La_0.44_Ca_0.56_Mn_0.95_O_2.40_, La_0.41_Ca_0.59_Mn_0.95_O_2.55_, and La_0.39_Ca_0.61_Mn_1_O_2.75_ for the vacuum, O_2_, and N_2_O depositions. It was further interesting to note that the growth orientation for the vacuum-grown film was (100), whereas the film orientations changed from (100) to (001) under reactive deposition conditions.

In the second example, we grew the thermoelectric (TE) material, Ca_3_Co_4_O_9_, as thin film on Pt-coated quartz with good TE properties before transferring these films as working pixels onto a flexible substrate [66]. The deposition was done from a stoichiometric target with λ = 248 nm, p_O2_ = 0.25 mbar, a laser fluence of 1.2 J/cm^2^ and a working distance of 3 cm. Under these conditions, films with a correct structure and composition of Ca_2.9_Co_4_O_9_, as determined by RBS, and desired TE properties were prepared. We further noticed that the relative ratio of O^−^ to O^+^ is largest (~ 6) at this position. Moving the TS distance to 4.5 cm, the film composition changed to Ca_2.9_Co_4_O_8.8_, the structure changed to a Ca_*x*_CoO_2_ phase and no O^−^ was measured at this position. Likewise, the TE properties have been poor. Both examples clearly demonstrate the supportive influence of negative species on the thin film growth in terms of crystalline quality, composition and physical properties.

In summary, we can conclude that the presence of negative plasma species can certainly be beneficial for the thin film growth and material properties. It is, however, in most cases not really possible to pinpoint their fluence directly.

### The source of oxygen in a film

When growing oxide thin films, it is of interest to know where the oxygen contributing to the overall film stoichiometry comes from. The three potential sources are the background gas, the target, or the substrate or a combination of the three sources. The way to discriminate the origin is to use ^18^O_2_ and substitute, e.g. ^16^O in the target material or the substrate and use mass spectrometry to investigate the differences between ^16^ and ^18^O.

Most of the oxygen in oxide films prepared in vacuum is provided by the target. This can clearly be seen when ablating a material in vacuum and analyse the oxygen content. One example was already presented in Sect. 3.5 when quoting the composition for a vacuum-grown film of La_0.6_Sr_0.4_MnO_3_ with La_0.44_Ca_0.56_Mn_0.95_O_2.40_. Another example is SrTiO_3_ where the oxygen content of the SrTiO_3−*x*_ thin films reaches values of ~ 2.5 [63]. Interesting is the fact that an oxygen content of 2.5 is typically encountered for a Brownmillerite phase. The oxygen-deficient SrTiO_3-*x*_ remains in a perovskite structure. The significant reduction in the oxygen is very typical for a vacuum ablation using a 248 nm or 193 nm laser. For a wavelength of 308 nm, also fully stoichiometric films have been prepared [52]. This indicates that the used wavelength seems to be important for the ablation process. One reason is that the KE of the ablated species becomes larger with increasing photon energy and therefore changes to the plume dynamics are expected [17]. Still, the majority of the oxygen in a film is from the target. This can be measured when preparing films from an isotop- exchanged La_0.6_Sr_0.4_Mn^16/18^O_3_ target [52, 60, 62]. In vacuum-ablated films, an approximate 50% exchanged ^18^O was confirmed in similar quantities. With increasing O_2_ background pressure, the ^18^O content in films decreased and was completely absent at the highest O_2_ deposition pressure. The reason for the continuous decline in ^18^O with increasing oxygen pressure is twofold. There is constant creation and dissociation of MO species during the time plasma species reach the target, and the oxygen is taken from the largest source available, the background. In addition, the amount of ^18^O from the target is finite and small compared to the amount of background oxygen available. The larger the background pressure becomes, the less likely it is to have ^18^O as a reaction partner. Therefore, most of the additionally supplied oxygen for a deposition at a higher deposition pressure is supplied by the background gas.

**Fig. 5:**
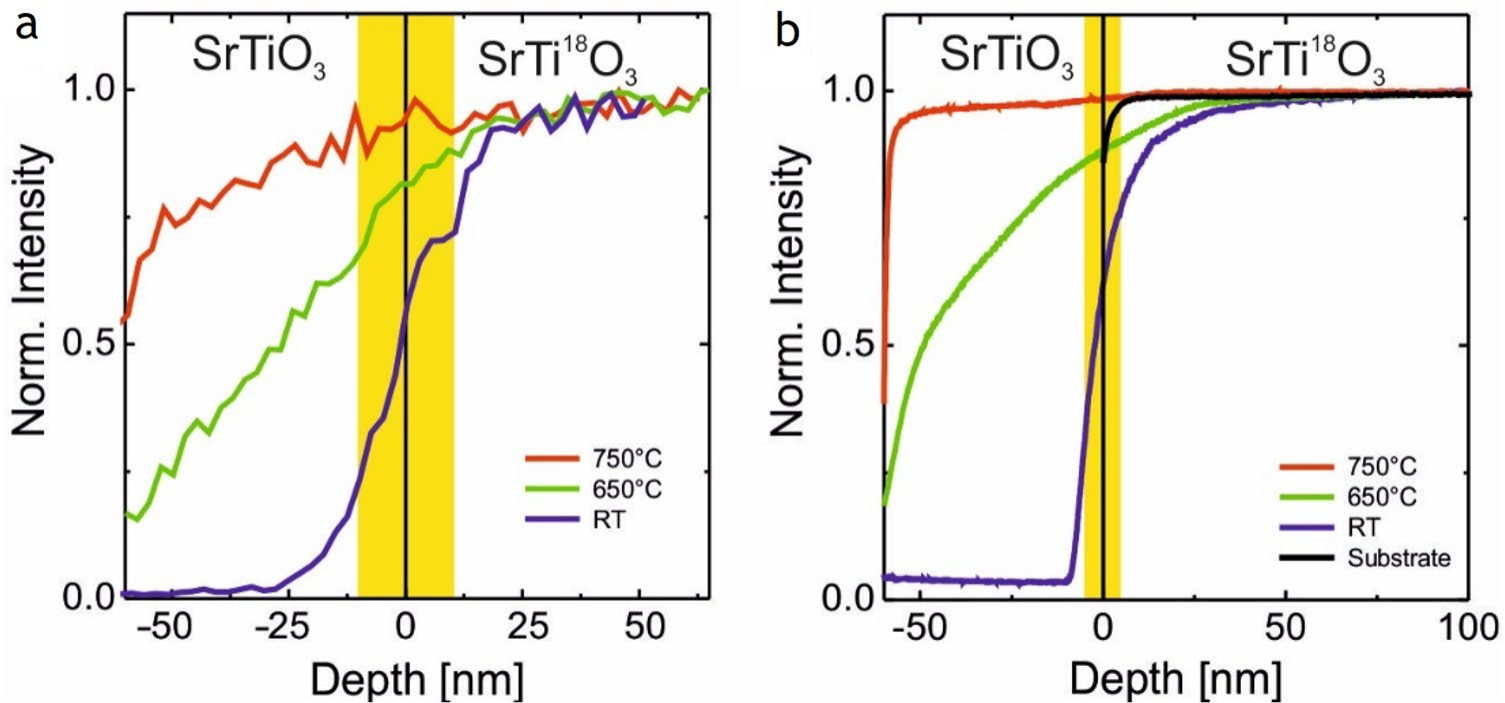
Normalized ^18^O depth profiles of  ≈ 60 nm thick SrTiO_3_ thin films grown at *p* = 1*.*5 × 10^–5^ mbar using PLD on ^18^O exchanged SrTiO_3_ substrates at three different temperatures: room temperature (blue), 650 ◦C (green), and 750 ℃ (red). The depth profiles have been measured with (a) ERDA and **b** SIMS [67, 68]. For comparison, a depth profile of a bare.^18^O exchanged substrate is shown (black). The yellow bar indicates the nominal width of the film–substrate interface as obtained from the depth resolution. Data from Ref. [67]

The substrate as a third component for the oxygen content of an oxide film cannot be neglected. This has been shown by SIMS and ERDA depth profiling of SrTiO_3_ and LaAlO_3_ thin films grown on ^18^O-exchanged SrTiO_3_ and LaAlO_3_ substrates [67, 68]. Figure 5 shows the ERDA and SIMS depth profiles of SrTiO_3_ thin films grown on SrTi^18^O_3_ at different deposition temperatures. Both measurements independently show that ^18^O is moved from the substrate to the film. For these SrTiO_3_ films, the oxygen supplied depends on the deposition temperature. We also observed that the exchange can take place at room temperature, in particular, if the growing film can take it. This is usually a very slow process and takes place over days to month. These experiments further indicate, that the initially formed film is oxygen deficient and a chemical gradient exists in favour of supplying oxygen via the substrate. It also means that the substrate must be able to supply oxygen. A class of materials, which is able to fulfil such a boundary condition, are ion conducting materials like yttria-stabilized zirconia oxide or SrTiO_3_. Other materials like LaAlO_3_ are likewise able to supply oxygen within limits. The extraction and exchange of oxygen from the substrate are also seen for as-grown SrTiO_3_ films prepared by rf sputtering and oxide MBE, in particular for films grown in a more reactive oxygen environment where the oxygen composition of the ^18^O-exchanged substrate changes during the growth of the film and ^18^O is partly replaced with ^16^O up to a depth of several tens of nm. Overall, these findings will probably be equally applicable for oxides used as substrate materials where oxygen diffusion and oxygen chemistry at elevated temperatures play a role [69].

### Plasma dynamics for target materials with a multi-elemental composition

**Fig. 6:**
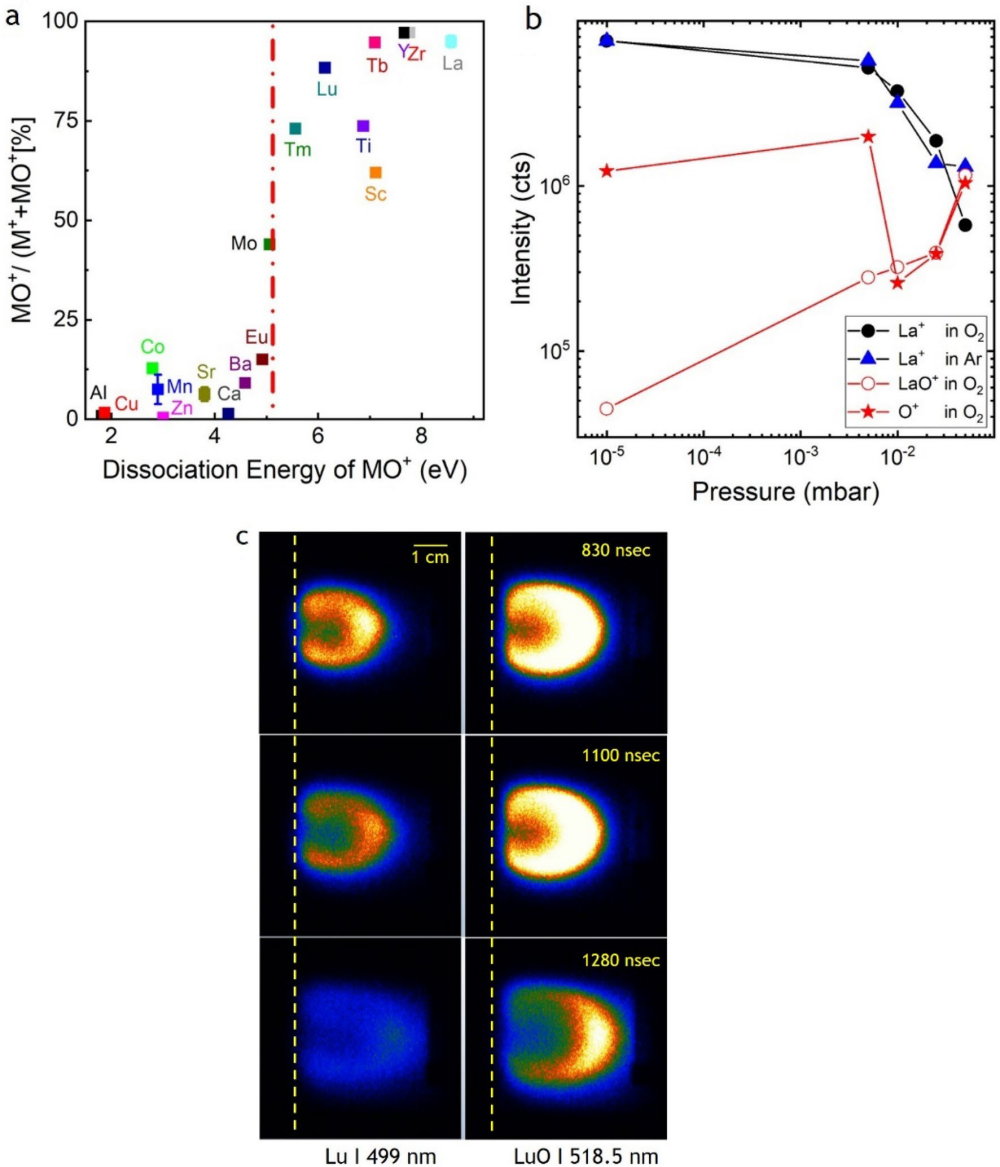
**a** The ratio of MO^+^/(M^+^ + MO^+^) vs. dissociation energy of MO^+^ species as determined at 0.15 mbar O_2_. The dashed line represents the dissociation energy of O_2_, *E*_diss_ (O_2_) = 5.12 eV [42, 60]; **b** Pressure dependence of La^+^, O^+^ and LaO^+^ in O_2_ and Ar. Figure from [42]. **c** Time-resolved plasma images from an LuMnO_3_ target show in the left column the Lu I emission at 499 nm and in the right column the LuO I at 518.5 nm [74]

The ablation from a multi-elementary target with three or more elements has its own dynamic in particular if there is a background gas involved. An idea has already been gained from the mass spectra shown in Fig. 4, and the relative mass ratio of the target species has a large influence (Fig. 3a). Additional examples are time and spatially resolved images of excited La I, Mn I, Ca I and O I species in Fig. 2a that show how fast these species move depending on their mass and how they are scattered. Following the mass spectra for the positive ions through the different O_2_ pressure ranges, the pattern as shown in Fig. 6a emerges at a typical deposition pressure of 0.15 mbar. MO species with a binding energy larger than 5 eV show a large MO^+^/(M^+^ + MO^+^) ratio (> 50%), whereas MO species with a binding energy smaller than 5 eV have correspondingly small MO^+^/(M^+^ + MO^+^) ratios. The dividing line is ~ 5 eV, which represents either the photon energy of the 248 nm laser or the dissociation energy of O_2_ (*E*_diss_ (O_2_) = 5.12 eV). At this O_2_ pressure, it is *E*_diss_ (O_2_) because the amount of MO^+^ species increases with increasing O_2_ pressure, which means that more of these species are created than dissociated [42, 60].

Measuring the KED of plasma species at different background pressures (Ar, O_2_), we note that the majority of slowed down species irrespective of the background gas are pushed to small kinetic energies, which strongly suggests that the expansion of M^+^ species with *E*_diss_ < *E*_diss_(O_2_) in O_2_ and Ar is mainly influenced by non-reactive collisions. Evaluating the total number of species, the number of M^+^ is approx. twice as big in O_2_ as compared to Ar. The observed difference is in parts the result of non-reactive collisions and includes in addition scattering events as a result of chemical reactions during the ablation in O_2_. This assumption is supported by the pressure dependence of the integrated area of O^+^ ions (Fig. 6b, red star). The number of O^+^ ions measured in vacuum from the KED correspond to the oxygen coming from the target, the same is largely correct for *p* = 1 × 10^–3^ mbar [62]. The strong dip at *p* = 1 × 10^–2^ mbar is the consequence of strongly scattered oxygen being the lightest element, whereas the recovery of numbers at larger pressures is the result of the background gas starting to be involved in the chemical reactivity of the plasma. This observation confirms previous experiments where most of the oxygen in *La*_*0.6*_*Sr*_*0.4*_*MnO*_*3*_ films deposited at a higher deposition pressure originates from the background gas [62]. As for La^+^, the corresponding Ar and O_2_ pressure dependencies show a decrease in the number of species with increasing pressure, whereas the number of LaO^+^ species increases over the same pressure range by almost two orders of magnitude. This indicates that La^+^ species are actively involved in the formation of LaO^+^ species with increasing O_2_ pressure a kinetic energy window of up to 5 eV to be the most effective energy regime to form LaO^+^ species [42].

The active participation of M^+^/MO^+^ species in the chemistry of the plasma plume can be visualized by time resolved imaging. This will be shown next for Lu and LuO. These species are easier to detect in emission with comparable emission lifetimes for both [70–73] and a binding energy of ~ 6.4 eV for LuO, which is similar to LaO (~ 8.4 eV). In Fig. 6c the expansion of Lu I and LuO I species 830, 1100, and 1280 ns after laser impact of a LuMnO_3_ target is measured in 5 × 10^–2^ mbar N_2_O with a fluence of *Φ* = 3.5 J/cm^2^ [18]. Using N_2_O and a higher fluence provides an overall more reactive atmosphere, making the conversion of Lu^+^ to LuO^+^ easier to visualize. Otherwise, the reaction dynamic in an oxygen or N_2_O background gas is similar. The intensity of the images shown in Fig. 6c ise scaled to the maximum intensity for the LuO I line at this pressure. The intensity scale for the ablation at 5 × 10^–2^ mbar is 10 × larger as compared to the ablation at 1.5 × 10^–3^ mbar, indicating a strongly enhanced scattering activity. The measured LuO I intensity distribution shows clearly, after 475 ns, a half-circe/half-moon distribution with high intensities in particular in the front regions of the plasma plume. With increasing time, the LuO I half-moon distribution becomes more extended and more intense, whereas the corresponding Lu I signal decreases within the same time window. After 1280 ns, the Lu I signal becomes faint and the majority of re-excitations for LuO I are located in the front of the plasma plume. Here, the fast Lu species in the plume scatter with the compressed cloud of N_2_O from the background gas. The volume covered by the "shell" distribution of LuO I increases overall and is visible in nearly all the areas between the target and mass spectrometer. The intensity is still comparably large, clearly outshining the Lu I emission. After 1825 ns, the LuO is still visible with the intensity maximum at the very front of the expending plasma, while an Lu I emission is almost not measurable.

This example for Lu/LuO shows at a constant background pressure the time evolution of the Lu I and LuO^+^ species [18]. With increasing time, the number of Lu I decreases, whereas the LuO I signal becomes progressively stronger indicting the conversion of Lu species into the thermodynamically more stable LuO. This behaviour mirrors the pressure evolution shown in Fig. 6b for the example of La/LaO [42]. Here, with increasing deposition pressure, the total number of La species measured decreases, whereas the total number of LaO species increases. Both processes indicate a chemical activity in the plasma plume.

What are the lessons learned from the presented experiments in particular for ns ablation as typically used for oxide thin film deposition? The main results are summarized as follows: in Fig. 3a, the strong relationship between the atomic mass ratios and the compositional deviations in the film; in Fig. 6a, the M^+^/MO^+^ ratio vs. dissociation energy where a separation line around 5 eV indicates that the stability of plasma species and hence the specific plasma species composition of a plume depends on parameters such as laser wavelength or dissociation energy of the background gas. The stability line further indicates that species with a binding energy larger than the dissociation energy and/or photon energy are preferentially formed if the opportunity arises and is certainly correct for a reactive atmosphere such as oxygen or N_2_O at a higher background pressure.

PLD is a deposition technique where the momentum transfer from the intense laser beam to the evaporated target material is a very dominant factor. As a result, light species are typically the fastest species and heavy species the slow ones in a plasma plume. On the other hand, light species are scattered more easily and hence they are distributed over a larger volume as compared to heavy species where the more forward directed character of the expanding plasma is preserved. Nevertheless, the velocity of these light species is significantly faster than the velocity of the heavy species reaching the substrate position. In a sense, most of these very fast species are lost in the formation of a film, because they often simply bounce off the substrate surface not taking part in the film formation. In parts, this is observed as a backflow of species after reaching the substrate position and partly the reason for a large deviation of the film composition as indicated by the findings in Fig. 3a. A solution is to moderate the kinetic energy of the very fast species either by the deposition pressure or to vary the TS distance or a combination of both. It would also mean that for a vacuum ablation, the very early arriving species with a very large KE contribute little or not at all to the creation of a growing film, whereas the later arriving species with an average smaller KE are mainly involved in the film formation. This would rationalize why very often a modest fluence between 1 and 2 J/cm^2^ is sufficient for the successful growth of oxide thin films and the laser fluence in general is an important, but not a strong parameter for the deposition of films. Fluence is usually correlated with the number of species removed from a target and their average KE, and the number of species removed will depend strongly on the absorption of the laser wavelength by the target material. If more species are removed, more scattering in a plume will take place and as a consequence the KE of species will be moderated more than in a less dense plume.

As a last point, slow plume species in a reactive background contribute to the formation of MO^+^ significantly more than faster species. This is reasonable, since the scattering (reaction) cross section of slow species is larger than for fast species and shown in ref [42] for La^+^/LaO^+^ (Fig. 6b). For a KE between 0 and 5 eV more than 90% of the measured LaO^+^ species have been formed, and between 5 and 15 eV 98% indicating that most LaO^+^ species have been formed within a kinetic energy window of 15 eV. This is the first time that a more quantitative analysis of KED confirmed what was expected: that the high scattering density of plume and background species is the driving parameter for the general chemistry in the laser-induced plume. Overall, the presented observations show that we gained an advanced understanding of pulsed laser ablation of oxide materials with a complex composition and developed some tools with predictive powers how to approach successfully the deposition of such system.

## Conclusions

In summary, we have shown that the time, space and species evolution of a laser-induced plasma are very complex, in particular if the ablation takes place from a multi-elemental target containing three or more elements. The maximum kinetic energies of ionic species in the laser-ablation plasma are strongly dependent on their masses, with higher masses yielding higher kinetic energies. In addition, the kinetic energies are fluence dependent [16, 75]. Adding O_2_ as background gas, there is a favourable energy window where plasma species reacted with the background O_2_, since a significant amount of MO^+^ species with low kinetic energies were detected with increasing pressure, as a result of the formation of the MO species. The preferred kinetic energy range of M species to form MO species in an expanding plasma plume, as a result of chemical reaction at a high O_2_ background pressure, is approximately up to 5 eV. We have shown further that the species composition of a plume can be correlated to the type of background gas used, and with increasing O_2_ background pressure, the number of MO species increases because more are created than dissociated. The plume composition and the corresponding film stoichiometry depend on the deposition pressure and is in addition fluence dependent. Moreover, there is a geometrical component of the laser beam profile, the flip-over or crowd effect, contributing to an anisotropy of the species distribution in a plume.

Negative ions in a plasma may have a positive effect on the growth properties of films, in particular if a more reactive background gas like N_2_O is used. Another important result is the identification of the oxygen sources contributing to the overall composition of an oxide film. Most oxygen is provided by the target for a vacuum ablation, and with increasing deposition pressure the background gas becomes the main oxygen source. The effect the background gas has on growth properties is more related to the species composition since the ratio of MO and M species arriving at a substrate will determine the chemistry to form the film.

We have also shown how to overcome the issue with the deposition of light elements not arriving stoichiometrically at the substrate. Here, the distance dependence seems to be as important as the pressure dependence to moderate *E*_*k*in_, which also means that some fast species are still needed to facilitate good crystalline growth.

## Supplementary Information

Below is the link to the electronic supplementary material.

Supplementary file1 (DOCX 1352 KB)

Supplementary file2 (GIF 1332 KB)

## Data Availability

The datasets generated during and/or analysed during the current study are available from the corresponding authors on reasonable request.
