# Supplementary material for: PLD plasma plume analysis: a summary of the PSI contribution
Source: Appl Phys A Mater Sci Process. 2023 Jan 21;129(2):138. doi: 10.1007/s00339-023-06408-4 (PMC9867658; doi:10.1007/s00339-023-06408-4)
Supplement: Supplementary file 1 — Supplementary file1 (DOCX 1352 KB) [file 339_2023_6408_MOESM1_ESM.docx]

Supplementary Information:

**PLD plasma plume analysis, a summary of the PSI contribution**

Christof W. Schneider^1^ & Thomas Lippert^1,2^

*^1^Laboratory for Multiscale Materials Experiments, Paul Scherrer Institute, 5232 Villigen PSI, Switzerland*

*^2^Department of Chemistry and Applied Biosciences, Laboratory of Inorganic Chemistry, ETH Zurich, Switzerland*

Figure S1


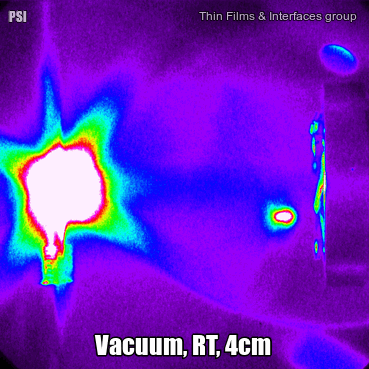


Figure S1: Video of the plasma expansion of silver in vacuum as recorded between the initial impact of the laser beam on the target and 10µsec with a time resolution of 500nsec for each frame. After approx. 2µsec a rebound of the impinging Ag species from the heater (TD-distance 4 cm) is observed. These rebounded species travel back even as far as the target.
