# Supplementary figures and images for: PLD plasma plume analysis: a summary of the PSI contribution
Source: Appl Phys A Mater Sci Process. 2023 Jan 21;129(2):138. doi: 10.1007/s00339-023-06408-4 (PMC9867658; doi:10.1007/s00339-023-06408-4)

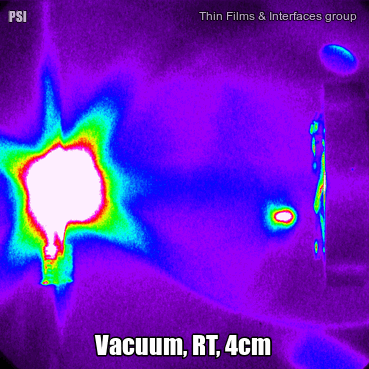

Supplement: Supplementary file 2 — Supplementary file2 (GIF 1332 KB) [file 339_2023_6408_MOESM2_ESM.gif]
